# Microshear Bond Strength of Composite Resin to Mineral Trioxide Aggregate and Potassium Nitrate‐Modified Polycarboxylate Cement at Two Time Points Using Different Adhesive Approaches: An In Vitro Study

**DOI:** 10.1002/cre2.70287

**Published:** 2026-01-09

**Authors:** Alireza Adl, Zahra Jowkar, Mahdi Zerafat, Fereshte Sobhnamayan

**Affiliations:** ^1^ Biomaterials Research Center, Department of Endodontics, School of Dentistry Shiraz University of Medical Sciences Shiraz Iran; ^2^ Oral and Dental Disease Research Center, Department of Operative Dentistry, School of Dentistry Shiraz University of Medical Sciences Shiraz Iran; ^3^ Department of Endodontics, School of Dentistry Shiraz University of Medical Sciences Shiraz Iran

**Keywords:** mineral trioxide aggregate, polycarboxylate cement, potassium nitrate, shear bond strength

## Abstract

**Objectives:**

This in vitro investigation evaluated the microshear bond strength (µSBS) of composite resin to mineral trioxide aggregate (MTA) and a modified polycarboxylate cement containing potassium nitrate (PCC/KNO_3_), both utilized as pulpotomy materials. The performance of two universal adhesives was compared, applied using self‐etch (SE) and etch‐and‐rinse (E&R) strategies.

**Materials and Methods:**

A total of 192 cylindrical acrylic specimens (2 cm height × 1 cm diameter) with a central cavity (4 mm diameter × 2 mm depth) were fabricated and filled with either MTA or PCC/KNO_3_ (*n* = 96 each). These were further divided into eight experimental subgroups (*n* = 12) based on adhesive type (All‐Bond Universal or Gluma Bond Universal), adhesive application method (SE or E&R), and storage duration (24 h or 7 days). After resin composite application, all specimens were stored in 100% humidity at 37°C for 24 h prior to µSBS testing. Data were analyzed using four‐way ANOVA and independent *t*‐tests (*α* = 0.05).

**Results:**

PCC/KNO_3_ exhibited significantly greater bond strength than MTA at both 24‐h (*p* < 0.001) and 7‐day (*p* = 0.030) intervals. Neither the adhesive type (*p* = 0.355) nor the application method (*p* = 0.358) significantly affected bond strength. Over time, µSBS values increased for MTA in some groups, while a significant decline was observed in most PCC/KNO_3_ subgroups.

**Conclusion:**

Due to its superior early bond strength and compatibility with immediate restoration, PCC/KNO_3_ shows potential as a viable alternative to MTA in vital pulp therapy, regardless of the adhesive strategy employed.

## 1 Introduction

Vital pulp therapy (VPT) or root canal treatment is commonly recommended when the dental pulp is exposed due to carious lesions, trauma, or iatrogenic factors. However, in immature permanent teeth with open apices and incompletely developed roots, conventional root canal treatment may not be the optimal approach (Ward 2002; Fuks 2008). In such cases, VPT is often preferred to promote continued root maturation and apical closure. Without this intervention, root formation is interrupted, resulting in thin dentinal walls that are structurally weak and vulnerable to fracture under masticatory forces (Fuks 2008; Vu et al. 2020). Preserving pulp vitality through procedures such as pulpotomy or direct pulp capping can help mitigate these complications.

Mineral trioxide aggregate (MTA) is a widely used bioactive material known for its biocompatibility and effectiveness in maintaining pulp and periapical tissue health (Rafter 2005; Shafiei et al. 2019). Its advantageous features include excellent sealing capability and notable antimicrobial activity (Torabinejad et al. 1995; Paños‐Crespo et al. 2021), and it has consistently shown high clinical success rates in vital pulp therapies (Kundzina et al. 2017; Mente et al. 2010; Torabinejad et al. 2018). Despite these benefits, MTA also has limitations, such as a prolonged setting time, relatively high cost, risk of tooth discoloration, and challenging handling properties (Torabinejad et al. 1995; Parirokh and Torabinejad 2010). Moreover, its bond strength to resin composite is initially low, especially during early setting stages, which often prevents immediate placement of definitive restorations (Alzraikat et al. 2016). Acid‐etching the MTA surface before restoration has also been reported to compromise its compressive strength and microhardness, further complicating early restorative procedures (Alzraikat et al. 2016).

Potassium nitrate (KNO_3_) has long been recognized as an effective desensitizing agent for managing dentin hypersensitivity (Hodosh 1974). Histological studies have demonstrated that polycarboxylate cement (PCC) can be successfully utilized as a direct pulp capping material (McWalter et al. 1973; Rajasekharan et al. 2014). The incorporation of 5% KNO_3_ into PCC to create a combined formulation (PCC/KNO_3_) has shown encouraging results in animal studies for treating dental trauma (Azimi et al. 2014). Additional research has confirmed its efficacy as a pulp capping agent, with the added benefit of reducing postoperative pain (Tsanova 2003). A clinical investigation further reported a 98% success rate in managing deep carious lesions without pulp exposure over a 4‐year period using this material (Hodosh et al. 1983). In another comparative study, PCC/KNO_3_ and MTA were both employed as pulpotomy agents in immature mandibular molars, with PCC/KNO_3_ emerging as a viable and cost‐effective alternative to MTA, offering easier handling characteristics (Ahmed et al. 2021).

Achieving optimal bond strength between restorative materials and the underlying substrate is a key determinant of long‐term success in VPT, as the durability of the restoration directly affects treatment longevity (Arandi 2023; Jowkar et al. 2020; Shafiei et al. 2020; Fattah et al. 2021). Despite significant progress in adhesive dentistry, maintaining a stable and durable bond to tooth structures continues to present clinical and material challenges (Shafiei et al. 2014, 2017, 2020; Jowkar et al. 2021; Shafiei and Memarpour 2009). Universal adhesives, also known as multi‐mode adhesives, represent the latest generation of bonding agents and are designed to function in a variety of clinical situations (Nozari et al. 2024). They combine essential adhesive components into a single bottle and can be used in either the self‐etch (SE) or etch‐and‐rinse (E&R) mode, providing greater flexibility during application (Nozari et al. 2024).

To date, no studies have specifically evaluated the microshear bond strength (µSBS) of composite resin to PCC/KNO_3_. Therefore, the present study was conducted to investigate and compare the µSBS of resin composite to PCC/KNO_3_ and MTA, utilizing two universal adhesive systems. Four null hypotheses were tested: (I) The type of cement (MTA or PCC/KNO_3_) would have no significant impact on µSBS; (II) the choice of universal adhesive would not influence µSBS; (III) the adhesive performance would be unaffected by the application mode (SE vs. E&R); and (IV) the timing of testing (24 h vs. 7 days) would have no effect on the µSBS to either cement.

## 2 Materials and Methods

### 2.1 Ethical Approval and Compliance

The manuscript of this laboratory study has been written according to Preferred Reporting Items for Laboratory studies in Endodontology (PRILE) 2021 guidelines (Nagendrababu et al. 2021). This in vitro study involved no human participants, animal subjects, or biological tissues. The research utilized five dental materials in total: Two base cements (MTA and PCC/KNO_3_), two universal adhesives (Gluma Bond Universal and All Bond Universal), and one composite resin (Filtek Z350 XT) to evaluate the µSBS of composite resin using different adhesive approaches. Ethical approval for the study protocol was obtained from the Ethics Committee of the School of Dentistry, Shiraz University of Medical Sciences (Approval ID: IR.SUMS.REC.1402.572). All procedures were performed in accordance with institutional guidelines and the ethical standards outlined in the Declaration of Helsinki.

### 2.2 Sample Size Calculation

The materials utilized in the research are listed in Table 1. Based on the study by (Palma et al. 2018), the minimum required sample size was calculated using G*Power software version 3.1 (Heinrich Heine University, Düsseldorf, Germany). With a significance level of *α* = 0.05 and a statistical power of 80%, a total of 192 specimens (12 per group) was determined as adequate.

**Table 1:** Materials used in this study.

Material (LOT number)	Type	Manufacturer	Composition	Application instructions
Gluma Bond Universal (N010617)	Universal adhesive	Heraeus Kulzer GmbH, Hanau, Germany	10‐methacryloyloxydecyl dihydrogen phosphate (MDP) phosphate monomer, 4‐methacryloxyethyl trimellitate anhydride (4‐META), dimethacrylate resin monomers, acetone, silane‐treated fillers, polymerization initiators, and silane coupling agents.	**Self‐etch (SE) mode:** 1. Apply adhesive on the entire surface with a microbrush and rub for 20 s. 2. Dry with gentle air spray for more than 5 s until fixed (no movement of adhesive) 3. Light‐cure for 10 s. **Etch‐and‐rinse (E&R) mode:** 1. Apply 37% phosphoric acid etchant on the set cement and allow 20 s 2. Rinse the surface with air/water spray for 30 s, and dry it with absorbent paper 3. Apply adhesive with a microbrush and rub for 20 s 4. Dry with gentle air spray for more than 5 s until fixed (no movement of adhesive) 5. Light‐cure for 10 s.
All‐Bond Universal (2500000674)	Universal adhesive	Bisco; Schaumburg, IL, USA	10‐methacryloyloxydecyl dihydrogen phosphate (MDP), bisphenol A glycidyl methacrylate (Bis‐GMA), hydroxyethyl methacrylate (HEMA), ethanol, water, photoinitiators, and stabilizers.	**Self‐etch (SE) mode:** 1. Apply 1–2 drops of adhesive in a well 2. Apply 2 separate layers and scrub for 10‐15 s on the surface. No curing is required between the two applications. 3. Evaporate excess solvent by air spray for a minimum of 10 s. Ensure no adhesive movement with a homogenous glossy appearance. 4. Light‐cure for 10 s **Etch‐and‐rinse (E&R) mode:** 1. Apply 37% phosphoric acid etchant on the set cement and allow 20 s. Rinse thoroughly. Remove excess water with a cotton pellet or high‐volume suction for 1–2 s such that the surface remains moist. 2. Apply adhesive as mentioned in SE mode.
Filtek ^MR^ Z350 XT (NF21629)	Composite resin	3M‐ESPE, St Paul, USA.	Silane‐treated ceramic fillers (silica and zirconia), Bis‐GMA (bisphenol A glycidyl methacrylate), Bis‐EMA (ethoxylated bisphenol A dimethacrylate), UDMA (diurethane dimethacrylate), TEGDMA (triethylene glycol dimethacrylate), polyethylene glycol dimethacrylate (PEGDMA), silane‐treated silica nanoparticles, silane‐treated zirconia nanoparticles, butylated hydroxytoluene (BHT), and pigments.	1. Apply composite resin in increments with > 2 mm thickness. 2. Light cure for 20 s
OrthoMTA (OMCE01D01)	Calcium silicate‐based cement	BioMTA, Seoul, Korea	Tricalcium silicate, dicalcium silicate, calcium carbonate, bismuth oxide, and trace amounts of calcium sulfate.	Mix cement powder with the required amount of distilled water to reach optimal consistency. The initial setting time of this cement is 3 h.
Polycarboxylate cement (10400515254)	Cement	Master‐Dent, Dental Technologies Inc., Lincolnwood, IL, USA	Zinc oxide, magnesium oxide, polyacrylic acid, water, stannous fluoride, and pigments.	Mix 100 g of powder with polyacrylic acid liquid in 1:1 ratio to obtain a putty‐like consistency with a working time of 1 min and 45 s.

### 2.3 Specimen Preparation

**Figure 1:**
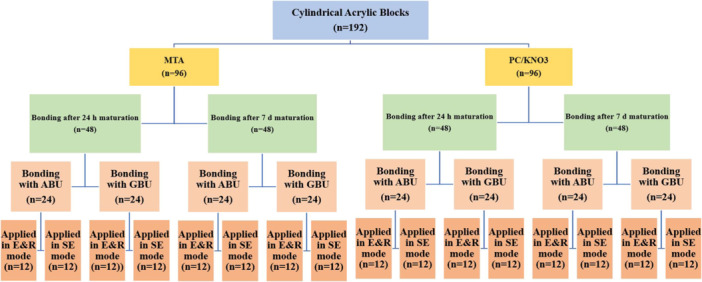
Acrylic specimens were filled with MTA or PCC/KNO₃ and allowed to mature for 24 h or 7 days. After each maturation period, specimens were bonded with composite using either All‐Bond Universal (ABU) or Gluma Bond Universal (GBU) in self‐etch (SE) or etch‐and‐rinse (E&R) mode. All specimens were tested for microshear bond strength (µSBS) 24 h after bonding, resulting in eight subgroups per cement type. MTA: mineral trioxide aggregate; PCC/KNO_3_: polycarboxylate cement with potassium nitrate.

Figure 1 illustrates the experimental grouping and procedures. A total of 192 cylindrical acrylic specimens (2 cm height × 1 cm internal diameter) were fabricated from acrylic resin (Acropars; Marlik Co., Tehran, Iran) using stainless steel molds. A central cavity (4 mm diameter × 2 mm depth) was drilled into each specimen. These samples were randomly assigned to two main groups (*n* = 96) and filled with either Ortho MTA (Bio MTA, Seoul, Korea), prepared according to the manufacturer's instructions, or PCC/KNO_3_. PCC/KNO_3_ was prepared by first finely grinding 5 g of potassium nitrate powder (Sigma‐Aldrich, St. Louis, MO, USA) using a mortar and pestle to ensure a uniform particle size. The ground KNO_3_ was then thoroughly blended with 95 g of PCC powder (Dentonics Inc., Monroe, NC, USA) in a dry state for 2 min to achieve a homogeneous mixture. This powder mixture was subsequently combined with polyacrylic acid liquid in a 1:1 ratio (powder:liquid) and manually mixed until a putty‐like consistency suitable for placement was obtained. The working time for this mixture was approximately 1 min and 45 s (Ahmed et al. 2021). After placement into the acrylic molds, both cements were allowed to set for approximately 10 min at room temperature to achieve initial hardening. For MTA samples, a moistened cotton pellet was placed over the surface to maintain hydration, whereas no cotton pellet was used for PCC/KNO_3_ samples, which set adequately under ambient conditions. All specimens were then sealed with a temporary restorative material (Cavisol; Golchai, Karaj, Iran) and stored at 37°C in 100% humidity for either 24 h or 7 days (*n* = 48 per interval). These storage durations represented the two time intervals for bonding and testing, as indicated in the study design (Figure 1). After the respective storage periods, the temporary material was carefully removed. Each main group was subdivided into four subgroups (*n* = 12) based on the adhesive system used—either All‐Bond Universal (ABU; Bisco, Schaumburg, IL, USA) or Gluma Bond Universal (GBU; Heraeus Kulzer GmbH, Hanau, Germany)—and the bonding mode applied (SE or E&R).

### 2.4 Composite Resin Application

**Figure 2:**
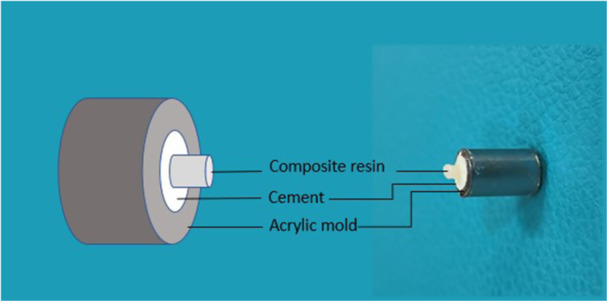
Illustration of specimen preparation: Placement of composite microcylinders on the cement‐filled acrylic molds prior to light curing.

For composite placement, polyvinyl chloride microtubes (0.7 mm internal diameter × 0.5 mm height) were positioned on the cement surface and filled with Filtek Z350 XT composite resin (3M‐ESPE, St. Paul, USA). The composite was light‐cured for 20 s using a curing unit (VIP Junior; Bisco, Schaumburg, IL, USA) operating at 600 mW/cm^2^ (Figure 2). Following adhesive application and composite bonding, all specimens were stored at 37°C in 100% humidity for an additional 24 h before µSBS testing. It should be noted that the pre‐bonding storage period for the cements was either 24 h or 7 days, depending on the experimental subgroup.

### 2.5 Microshear Bond Strength Testing

**Figure 3:**
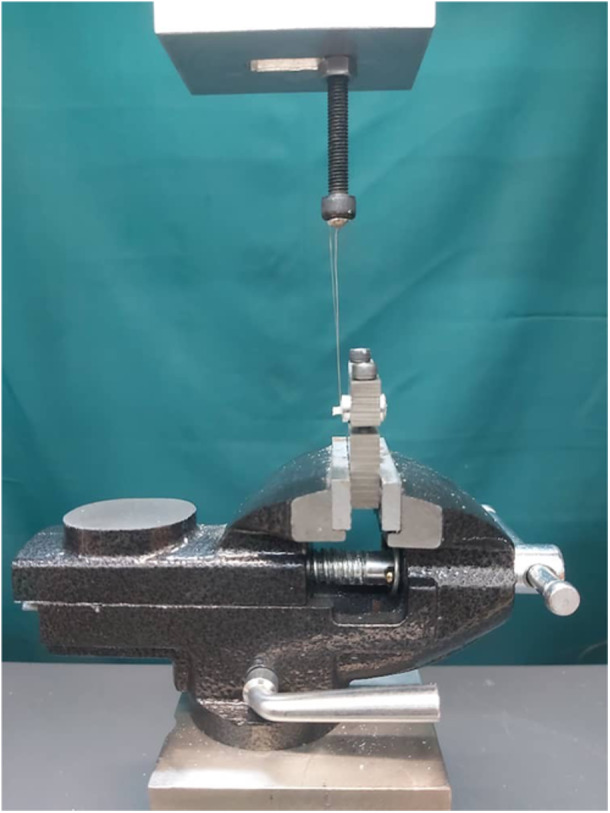
Positioning of a bonded specimen under the universal testing machine for microshear bond strength testing. A stainless‐steel ligature wire is wrapped around the base of the composite micro‐cylinder to apply shear force.

Bonding of composite resin was performed at two cement maturation intervals: 24 h and 7 days after cement placement. For all specimens, µSBS testing was conducted 24 h after bonding, ensuring consistent post‐bonding evaluation across all subgroups. Figure 1 illustrates the experimental grouping, adhesive application modes, and bonding timeline. Each sample was mounted in a universal testing machine (Instron, Z020, Zwick Roell, Germany) for µSBS evaluation (Figure 3). A shear force was applied to the composite‐cement interface at a crosshead speed of 0.5 mm/min until failure occurred. The µSBS (in MPa) was calculated by dividing the peak force at failure (F, in Newtons) by the bonded surface area (A, in mm^2^), as shown in the following formula:

$$\mu \text{SBS}=\frac{F}{A}$$

### 2.6 Failure Mode Analysis

Post‐test failure modes were examined under a stereomicroscope (Carl Zeiss Inc., Oberkochen, Germany) at 40× magnification, and categorized as follows (Altunsoy et al. 2015):

- Adhesive failure: Separation at the adhesive interface with visible adhesive on the cement surface.
- Cohesive failure: Fracture within the composite or cement itself.
- Mixed failure: A combination of adhesive and cohesive failure in the composite and/or cement.

#### 2.6.1 Statistical Analysis

Data normality was confirmed using the Shapiro–Wilk test. A four‐way ANOVA was conducted to evaluate the effects of cement type, adhesive type, application method, and storage time on µSBS. Independent *t*‐tests were subsequently used for subgroup comparisons. All statistical analyses were performed using SPSS version 20 (IBM Corp., Chicago, IL, USA), with significance set at *p* < 0.05.

**Figure 4:**
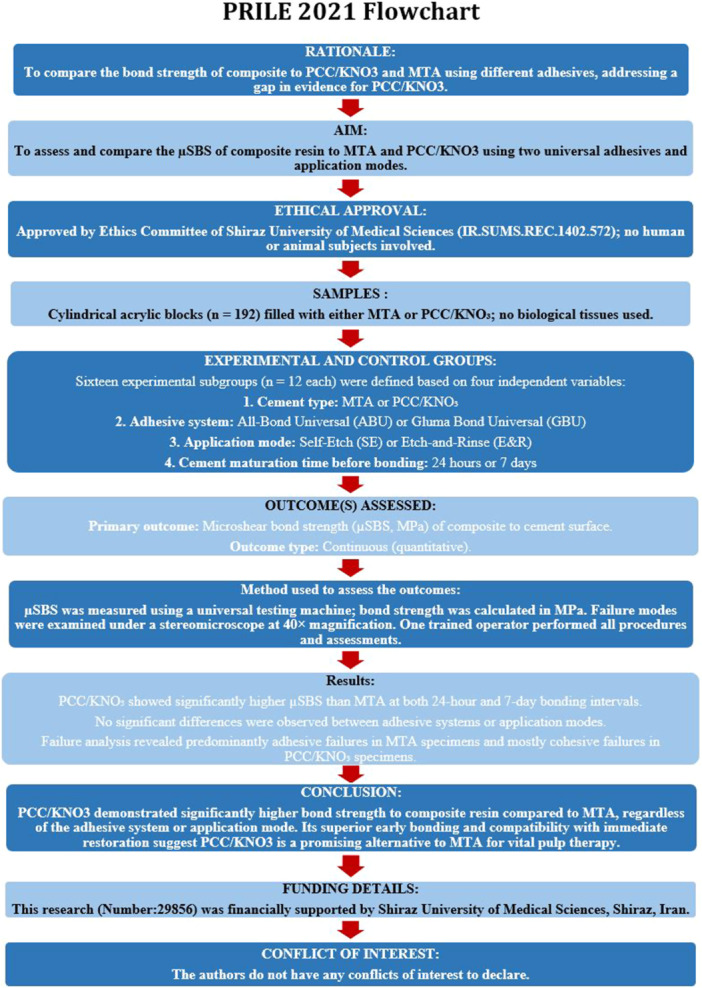
PRILE 2021 flowchart illustrating the study design, sample preparation, group allocation, adhesive application modes, and testing timeline used in the microshear bond strength evaluation of composite resin to MTA and PCC/KNO_3_. µSBS, microshear bond strength; MTA, mineral trioxide aggregate; PCC/KNO_3_, potassium nitrate‐modified polycarboxylate cement.

The detailed study design, sample preparation, group allocation, adhesive application protocols, and testing timeline are summarized in the PRILE 2021 flowchart (Figure 4), in accordance with the Preferred Reporting Items for Laboratory studies in Endodontology guidelines (Nagendrababu et al. 2021).

## 3 Results

**Figure 5:**
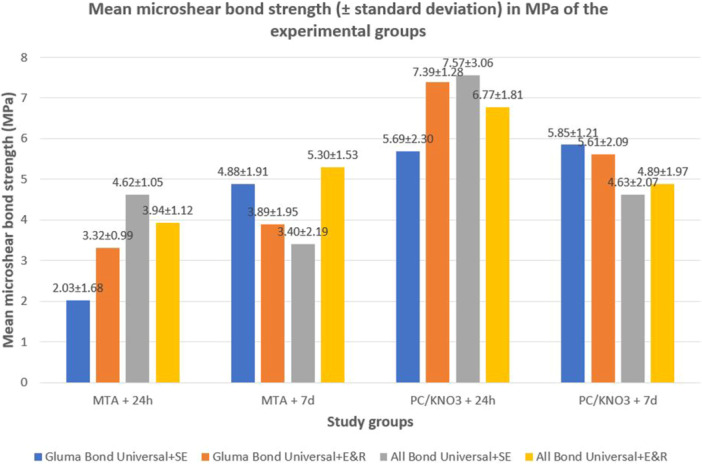
Bar graph showing the mean microshear bond strength (µSBS) values in MPa for all experimental groups at 24‐h and 7‐day intervals. µSBS: microshear bond strength; d: day; E&R: etch‐and‐rinse; h: hour; MPa: megapascal; MTA: mineral trioxide aggregate; PCC/KNO_3_: polycarboxylate cement with potassium nitrate; SE: self‐etch.

Figure 5 illustrates a bar graph depicting the mean µSBS values, along with standard deviations (SD), for all test groups. The measurements are expressed in megapascals (MPa).

It should be noted that the reported time intervals (24 h and 7 days) refer to the cement maturation period prior to bonding, not the storage time after bonding. For all subgroups, the composite resin was bonded at either 24 h or 7 days after cement placement, and µSBS testing was conducted 24 h after bonding.

A four‐way ANOVA indicated statistically significant interactions between the variable time and each of the three remaining factors—cement type, adhesive system, and application technique (*p* < 0.05). Consequently, pairwise comparisons were performed at each time point using independent *t*‐tests, as summarized in Table 2. The results demonstrated that irrespective of the adhesive used or its application mode, the µSBS of composite resin to PCC/KNO_3_ was significantly greater than that to MTA at both 24‐h (*p* < 0.001) and 7‐day (*p* = 0.017) cement maturation intervals.

**Table 2:** Mean (SD) microshear bond strength (MPa) according to cement type, adhesive type, and application method at 24 h and 7 d.

Variables		24 h	7 d
Cement type	MTA	3.60 ± 1.45	4.65 ± 1.83
	PCC/KNO_3_	7.21 ± 2.02	5.55 ± 1.63
	*p* value	< 0.001*	0.017*
Adhesive type	All‐Bond Universal	5.85 ± 2.38	4.99 ± 1.74
	Gluma Bond Universal	4.86 ± 2.58	5.19 ± 1.83
	P value	0.060	0.065
Method of application	E&R	5.40 ± 2.19	5.15 ± 1.78
	SE	5.33 ± 2.85	5.05 ± 1.80
	*p* value	0.889	0.787

Abbreviations: d, day; E&R, etch‐and‐rinse; h, hour; MTA, mineral trioxide aggregate; PCC/KNO_3_, polycarboxylate cement with potassium nitrate; SE, self‐etch.

* Indicates significant *p*‐values (*p* < 0.05).

No significant differences in µSBS were detected between the two universal adhesives—ABU and GBU—regardless of cement type or maturation time. Specifically, *p*‐values were 0.060 and 0.065 for the 24‐h and 7‐day cement maturation intervals, respectively. Similarly, the adhesive application method (SE vs. E&R) did not yield statistically significant differences at either time point (*p* = 0.889 at 24 h; *p* = 0.787 at 7 days).

The influence of cement maturation time on bond strength was further analyzed within all eight experimental subgroups (Table 3). In the MTA groups, µSBS generally increased or remained stable over time, whereas PCC/KNO_3_ groups tended to show reduced bond strength at longer maturation periods.

**Table 3:** Mean (SD) microshear bond strength (MPa) and *t*‐test results for the effect of time within each experimental subgroup.

Cement	Adhesive	Method	24 h	7 d	*p* value
MTA	All‐Bond Universal	E&R	3.94 ± 1.12	5.30 ± 1.53	0.022*
		SE	4.62 ± 1.05	3.93 ± 1.99	0.310
	Gluma Bond Universal	E&R	3.32 ± 0.99	4.15 ± 1.82	0.189
		SE	2.30 ± 1.72	5.08 ± 1.87	0.002*
PCC/KNO_3_	All‐Bond Universal	E&R	7.09 ± 1.52	5.52 ± 1.41	0.025*
		SE	8.04 ± 2.72	5.16 ± 1.83	0.011*
	Gluma Bond Universal	E&R	7.39 ± 1.28	5.61 ± 2.09	0.020*
		SE	6.21 ± 2.17	5.85 ± 1.21	0.629

Abbreviations: d, day; E&R, etch‐and‐rinse; h, hour; MTA, mineral trioxide aggregate; PCC/KNO_3_, polycarboxylate cement with potassium nitrate; SE, self‐etch.

* Indicates significant *p*‐values (*p* < 0.05).

**Figure 6:**
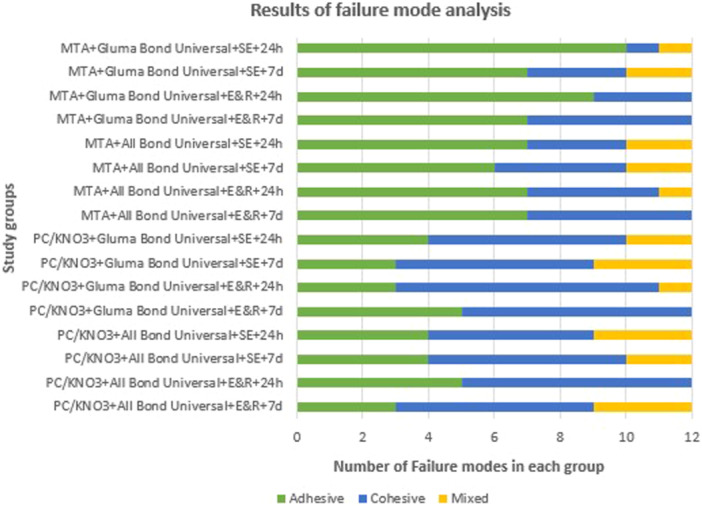
Distribution of failure modes observed in the study groups. d: day; E&R: etch‐and‐rinse; h: hour; MTA: mineral trioxide aggregate; PCC/KNO_3_: polycarboxylate cement with potassium nitrate; SE: self‐etch.

Figure 6 summarizes the analysis of failure modes across groups. The MTA‐based specimens predominantly exhibited adhesive failures, whereas PCC/KNO_3_ samples more frequently failed cohesively within the material.

## 4 Discussion

This study aimed to evaluate the µSBS of composite resin to two pulpotomy materials—MTA and PCC/KNO_3_—using two commercially available universal adhesives. The findings revealed that PCC/KNO_3_ provided significantly greater µSBS than MTA at both 24‐h and 7‐day intervals, leading to the rejection of the first and fourth null hypotheses. Conversely, no meaningful differences were observed between the adhesive systems used (ABU and GBU) or between their application strategies (SE vs. E&R), thus supporting the second and third hypotheses.

In the present study, µSBS was evaluated as a function of cement type, adhesive system, application mode, and cement maturation time. Importantly, all µSBS measurements were performed 24 h after composite bonding, while the reported time points (24 h and 7 days) represent the cement maturation periods prior to bonding. This design allowed assessment of how cement maturation influences bonding performance. Overall, MTA exhibited stable or increased µSBS with longer maturation, likely due to continued hydration and microstructural stabilization, whereas PCC/KNO_3_ tended to show reduced µSBS over time, possibly related to surface dissolution or saturation. This clarification of timing enables a more accurate interpretation of the individual and combined effects of the studied variables on bond strength.

The mechanism of adhesion between composite resin and calcium silicate‐based materials has been explored in previous literature. Functional monomers present in universal adhesives are known to establish chemical bonds with various ions, including calcium, silicon, zirconium, and aluminum, which are integral components of such cements (Deepa et al. 2016; Singla and Wahi 2020). Moreover, recent evidence suggests that these monomers, particularly 10‐MDP, also demonstrate a high affinity for zinc, forming stronger bonds with zinc ions than with calcium (Feitosa et al. 2014). Given that PCC/KNO_3_ is zinc‐rich, its superior bond performance in this study may be attributed to the enhanced chemical interaction between zinc and the adhesive monomers.

Surface characteristics also play a crucial role in bond strength. The roughness and microtopography of the substrate can significantly influence adhesive retention (Ginebra et al. 1999; Shin et al. 2014). Although no direct comparison of surface morphology between MTA and PCC/KNO_3_ has been conducted, the improved bond strength to PCC/KNO_3_ may, in part, result from a surface structure that promotes better micromechanical interlocking.

In this investigation, both ABU and GBU were employed as universal adhesives for bonding to MTA and PCC/KNO_3_. ABU, categorized as an ultra‐mild adhesive with a pH of 3.2, contains methacryloyloxydecyl dihydrogen phosphate (MDP‐10), which facilitates limited demineralization of calcium‐based substrates like MTA and allows for deep monomer infiltration into the surface structure (Choi et al. 2017).

On the other hand, GBU is considered a strong adhesive (pH 1.8) and contains 10‐MDP as its primary functional monomer, along with 4‐methacryloxyethyl trimellitic anhydride (4‐META). 10‐MDP chemically interacts with calcium (e.g., in MTA), forming MDP‐calcium salts that precipitate onto the substrate surface. It can also react with zinc ions (e.g., in PCC/KNO_3_), forming MDP‐zinc phosphate deposits (Carrilho et al. 2019; Feitosa et al. 2014). 4‐META is expected to form similar ionic bonds with calcium and potentially zinc, although direct evidence for its interaction with zinc is currently lacking (Kalchinov et al. 2025). These precipitates help form water‐resistant nanolayers and support both chemical and micromechanical adhesion, resulting in the formation of a hybrid bonding layer (Van Meerbeek et al. 2011; Sanabe et al. 2009).

No statistically significant differences in µSBS were observed between ABU and GBU, irrespective of cement type, cement maturation period, or adhesive application mode in the present study. This observation aligns with the results reported by Kalyoncuoğlu et al. (2021) who found that variations in the pH of universal adhesives did not markedly influence the shear bond strength to calcium silicate‐based cements when bonded to resin composites. Their study suggests that lowering the pH does not necessarily enhance micromechanical retention. Despite their differing acidity, both ABU and GBU share similar adhesive chemistry, notably the presence of MDP‐10 (in both systems) and 4‐META (exclusively in GBU), which can interact chemically with calcium and zinc ions present in MTA and PCC/KNO_3_. These interactions result in the formation of stable nanolayers composed of calcium or zinc salts, reinforcing the chemical component of the hybrid bond (Kalyoncuoğlu et al. 2021). This molecular interaction explains the comparable µSBS values achieved with both adhesives in the current study.

Furthermore, the adhesive application mode (SE or E&R) did not significantly affect µSBS for either cement at any maturation time. This outcome is consistent with prior investigations that reported no substantial influence of application mode on bond strength when universal adhesives were used on calcium‐containing substrates (Hanabusa et al. 2012; Tyagi et al. 2016; Hashem et al. 2014; Marchesi et al. 2014; Sismanoglu et al. 2022). One likely explanation is that both MTA and PCC/KNO_3_ are calcium‐rich, and both ABU and GBU contain functional acidic monomers (10‐MDP and 4‐META) that can chemically interact with calcium ions regardless of the etching step, ensuring sufficient bond formation in both SE and E&R modes.

Cement maturation time significantly influenced µSBS, with distinct effects observed for the two materials evaluated in this study. Overall, PCC/KNO_3_ demonstrated significantly higher µSBS than MTA when bonding was performed after both 24‐h and 7‐day cement maturation periods, indicating superior early bonding performance to composite resin. Because all specimens were tested 24 h after bonding, the observed differences in µSBS can be attributed to cement maturation rather than post‐bonding storage. However, the two cements exhibited different time‐dependent behaviors. In MTA‐based specimens, µSBS generally increased or remained stable when bonding was delayed to 7 days, suggesting that ongoing hydration and microstructural maturation enhance both chemical and micromechanical adhesive interactions. In contrast, PCC/KNO_3_ tended to show reduced µSBS with longer maturation, with several subgroups exhibiting significant declines, while others remained unchanged, indicating that prolonged maturation may negatively affect surface characteristics relevant to bonding.

These time‐dependent trends can be explained by differences in the maturation processes and surface chemistry of the two materials. In MTA, continued hydration and crystal growth during the first week likely contribute to microstructural stabilization and improved bonding potential (Altan and Tosun 2016; Karunakaran et al. 2025). Conversely, the reduction in bond strength observed with PCC/KNO_3_ may be associated with early surface dissolution or saturation, which limits further interaction with functional adhesive monomers (Heboyan et al. 2023). These findings are consistent with previous reports showing that functional monomers interact differently with calcium‐ and zinc‐containing cements over time, underscoring the importance of considering cement maturation when planning adhesive procedures (Carrilho et al. 2019; Feitosa et al. 2014; Toledano et al. 2022).

From a clinical standpoint, the findings suggest that when using MTA as a pulpotomy material, postponing the placement of the final composite restoration for up to 7 days after cement placement may enhance bond integrity. In contrast, PCC/KNO_3_ exhibited higher bond strength when the composite was applied after 24 h of setting. These results indicate that the degree of cement maturation prior to bonding significantly influences the µSBS.

The lower µSBS values observed when bonding was performed after 7 days of PCC/KNO_3_ maturation may be attributed to changes in the cement's setting characteristics. In its early stage (after 24 h), PCC/KNO_3_ exhibits higher acidity and solubility, producing a more irregular and porous surface that favors micromechanical adhesion. As the material matures, solubility decreases and the surface becomes smoother and less retentive, which may reduce micromechanical interlocking and explain the lower bond strength observed at 7 days compared with 24 h (Panahandeh et al. 2015).

MTA, on the other hand, is known for its porous structure and slow setting reaction, involving the formation of a calcium silicate hydrate gel. This maturation process strengthens the material's bulk over time, making it more capable of withstanding the contraction stresses associated with composite polymerization. Earlier reports indicate that light‐curing–induced shrinkage stress can compromise the structural integrity of freshly placed MTA (Singla and Wahi 2020). Moreover, the material's alkalinity during initial setting may be partially neutralized by the acidic nature of adhesive systems, further weakening early bond strength (Hashem et al. 2014).

The higher µSBS observed when bonding was performed after 7 days of MTA maturation aligns with the findings of (Atabak et al. 2012), who recommended delaying bonding procedures for at least 96 h. Similarly, (Keerthivasan et al. 2023) reported enhanced bonding performance with extended MTA setting time.

However, differing conclusions were drawn by (Tsujimoto et al. 2013), who observed that immediate placement of composite over unset MTA can be effective when using adhesive systems. This discrepancy may stem from differences in study design, as Tsujimoto et al. (2013) focused on interfacial gap formation rather than direct bond strength, as measured in the present investigation.

Failure mode analysis further supports the bond strength outcomes. In MTA groups, adhesive failure was the predominant mode, while PCC/KNO_3_ specimens more frequently exhibited cohesive failures within the material. These results are in agreement with those of (Fatima et al. 2024), who noted that higher bond strengths are typically associated with cohesive or mixed failure patterns, whereas lower bond strengths tend to result in adhesive failures.

Despite the valuable insights provided by this study, some limitations should be acknowledged. Only two universal adhesives were examined, which may not comprehensively reflect the performance variability among the wide array of systems currently available on the market. Future research should incorporate a broader selection of adhesives, particularly those with diverse pH profiles, to better understand how acidity influences bond strength. Additionally, µSBS was assessed after short‐term water storage; thus, further investigations are necessary to evaluate the durability of the adhesive interface over extended periods. Most importantly, clinical trials are essential to confirm the in vivo efficacy and reliability of PCC/KNO_3_ as a novel pulpotomy material and to validate its long‐term clinical behavior.

## 5 Conclusion

Within the limitations of this in vitro study, PCC/KNO_3_ exhibited significantly greater µSBS to composite resin than MTA, independent of the adhesive system or its mode of application. The favorable early bonding performance of PCC/KNO_3_, observed when bonding was performed after 24‐h and 7‐day maturation periods, suggests that this material may serve as a clinically viable alternative to MTA for both immediate and delayed restorations in VPT.

## Author Contributions

**Alireza Adl:** conceptualization, data curation, formal analysis, funding acquisition, investigation, methodology, project administration, resources, software, supervision, validation, visualization, roles/writing – original draft, writing – review and editing. **Zahra Jowkar:** conceptualization, data curation, formal analysis, funding acquisition, investigation, methodology, project administration, resources, software, supervision, validation, visualization, roles/writing – original draft, writing – review and editing. **Mahdi Zerafat:** conceptualization, data curation, methodology, supervision, writing – review. **Fereshte Sobhnamayan:** conceptualization, data curation, methodology, supervision, writing – review.

## Conflicts of Interest

The authors declare no conflicts of interest.

## Data Availability

The data that support the findings of this study are available from the corresponding author upon reasonable request.
